# Is supported self-management for depression effective for adults in community-based settings in Vietnam?: a modified stepped-wedge cluster randomized controlled trial

**DOI:** 10.1186/s13033-020-00342-1

**Published:** 2020-02-12

**Authors:** Jill K. Murphy, Hui Xie, Vu Cong Nguyen, Leena W. Chau, Pham Thi Oanh, Tran Kieu Nhu, John O’Neil, Charles H. Goldsmith, Nguyen Van Hoi, Yue Ma, Hayami Lou, Wayne Jones, Harry Minas

**Affiliations:** 1 Department of Psychiatry, Faculty of Medicine, University of British Columbia, Mood Disorders Centre, 2255 Westbrook Mall, Vancouver, BC V6T 2A1 Canada; 2 Faculty of Health Sciences, Simon Fraser University, 8888 University Drive, Burnaby, BC V5A 1S6 Canada; 3 Institute of Population, Health and Development, 132/18 Hoa Bang Street, Cau Giay, Hanoi, 122667 Vietnam; 4 Centre for Applied Research in Mental Health & Addiction, Faculty of Health Sciences, Simon Fraser University, 515 West Hastings Street, Vancouver, BC V6B 5K3 Canada; 5 Ministry of Labour, Invalids and Social Affairs, 12 Ngo Quyen Street, Hoan Kiem District, Ha Noi, 159999 Vietnam; 6 BC Centre for Excellence in HIV/AIDS, 608-1081 Burrard Street, Vancouver, BC V6Z 1Y6 Canada; 7 Melbourne School of Population and Global Health, University of Melbourne, 207 Bouverie Street, Carlton, VIC 3053 Australia

**Keywords:** Task-sharing, Depression, Supported self-management, Vietnam

## Abstract

**Background:**

This study tested the effectiveness of a supported self-management (SSM) intervention to reduce symptoms of depression among adults compared with enhanced treatment as usual in community-based and primary care settings in Vietnam.

**Methods:**

The cluster randomized trial included 376 adults in 32 communes in eight provinces. Eligible participants scored > 7 on the SRQ-20 depression scale. Patients with severe symptoms were excluded and referred to tertiary care. Randomization took place at the commune level. The immediate intervention group included 16 communes with 190 participants and the delayed group included 16 communes with 186 participants. Participants in communes randomized to the immediate intervention group received a two-month course of SSM, consisting of a workbook and supportive coaching. Those in communes randomized to the delayed group received enhanced treatment as usual and, for ethical purposes, received the SSM intervention after 4 months. The primary outcome is the effect of SSM on reduction in depression scores as indicated by a reduced proportion of participants with SRQ-20 scores > 7 at 2 months after commencement of SSM intervention. Blinding was not possible during intervention delivery but outcome assessors were blinded. Analysis was intention-to-treat.

**Results:**

At 2 months, 26.4% of the intervention group and 42.3% of the delayed group had SRQ-20 scores > 7. The adjusted odds ratio of having depression between the intervention and control was 0.42 (p < 0.0001), 95% CI (0.28, 0.63). Receiving the intervention thus reduces the odds of having depression by 58%, compared with receiving the control after 2 months of treatment. No adverse events were reported.

**Conclusions:**

Results suggest that SSM is effective for decreasing depression symptoms among adults in community-based settings in Vietnam.

*Trial Registration* This trial is registered at ClinicalTrials.gov, number NCT03001063.

## Background

Unipolar depression is one of the leading contributors to the global burden of disease, with the highest percentage of the burden found in low and middle-income countries (LMICs) [1]. There is limited epidemiological evidence about depression in Vietnam, but existing studies suggest that prevalence is similar to global rates [2]. In Vietnam, like in many LMICs, a shortage of mental health specialists and very limited mental health services in general health and primary care settings have contributed to a critical gap in depression care. Services for depression are almost entirely unavailable in Vietnam [2], except in a few tertiary psychiatric hospitals.

Task-sharing models, wherein non-specialist providers deliver psychosocial interventions, have been recommended as an essential component of mental health care delivery in LMICs, while the importance of delivering services in the community has also been acknowledged as a means of improving access to care [3, 4]. Non-specialist providers, using evidence-based interventions and training packages, are recommended for delivering care in low-resource settings due to their affordability, sustainability and established relationships with the community [5], which supports trust building and reduces barriers to help-seeking [6]. The government of Vietnam has, in recent years, prioritized the enhancement of community-based care for depression, to be delivered through the health and social services sectors [2]. Supported-self management (SSM) for depression, a psychosocial intervention using a task-sharing approach and based on cognitive behavioural therapy (CBT) principles [7], can be delivered by non-specialist providers in primary care and community-based settings and has been shown to be acceptable and feasible for use in Vietnam [8].

This study tested the hypothesis that an SSM intervention for depression offered in community-based settings via task-sharing in Vietnam would decrease probable caseness of depression among adults compared with treatment as usual.

## Methods

### Study design

**Fig. 1:**
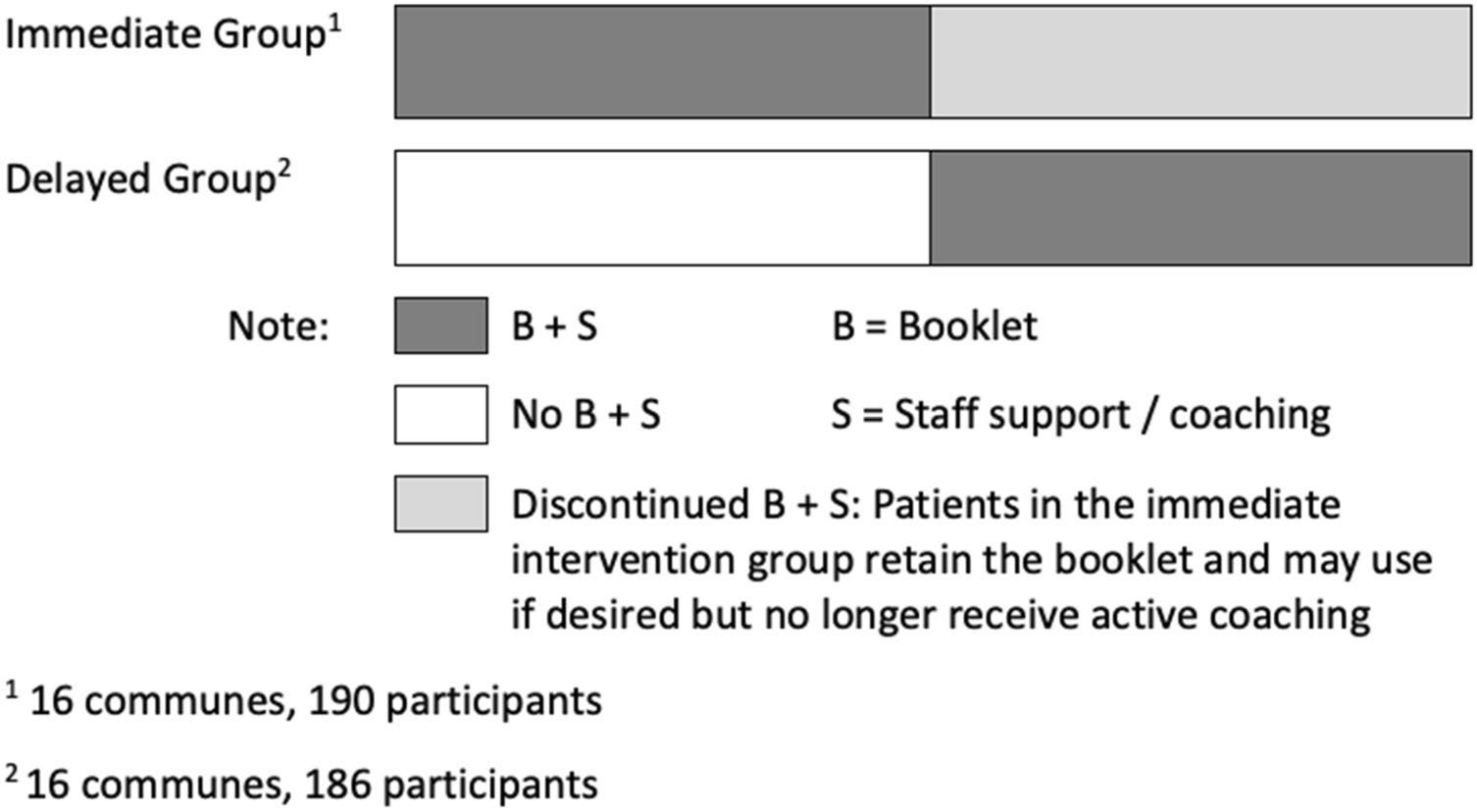
Study design

Using a cluster-randomized, modified stepped wedge controlled trial design (Fig. 1), the effectiveness of an SSM intervention to treat mild to moderate depression was tested among adults in community-based settings in Vietnam. Data collection took place between July 2016 and November 2017 in eight provinces across Vietnam (Tables 1 and 2). In each province, two districts and two communes (municipal subdivisions) within each district were randomly selected. Data collection took place in a total of 32 communes. Randomization occurred at the commune level, with communes assigned to receive the SSM intervention or the control condition in Period 1 of the study (Fig. 1).

**Table 1:** MAC-FI study sample by province, district and commune for Period 1

Province District *Communes*	Eligible No	Consent at baseline n1 (n1/n0*100%)	Follow-up 1 (after 1 month) n2 (n2/n1*100%)	Follow-up 2 (after 2 months) n3 (n3/n1*100%)
BEN TRE				
Ben Tre				
Son Dong (intervention)	31	29 (93.6)	28 (96.6)	27 (93.1)
Phuong 6 (delay)	9	3 (33.3)	3 (100.0)	2 (66.7)
Total n (%)	40	32 (80.0)	31 (96.9)	29 (90.6)
Giong Trom				
Phuoc Long (intervention)	15	12 (80.0)	9 (75.0)	11 (91.7)
Long My (delay)	10	9 (90.0)	3 (33.3)	2 (22.2)
Total n (%)	25	21 (84.0)	12 (57.1)	13 (61.9)
DA NANG				
Hoa Vang				
Hoa Nhon (intervention)	18	8 (44.4)	5 (62.5)	8 (100.0)
Hoa Tien (delay)	23	17 (73.9)	12 (70.6)	12 (70.6)
Total n (%)	41	25 (61.0)	17 (68.0)	20 (80.0)
Thanh Khe				
Chinh Gian (intervention)	17	16 (94.1)	12 (75.0)	13 (81.3)
Thanh Khe Tay (delay)	16	8 (50.0)	4 (50.0)	2 (25.0)
Total n (%)	33	24 (72.7)	16 (66.7)	15 (62.5)
KHANH HOA				
Dien Khanh				
Dien An (intervention)	12	9 (75.0)	6 (66.7)	5 (55.6)
Dien Dien (delay)	8	8 (100.0)	6 (75.0)	7 (87.5)
Total n (%)	20	17 (85.0)	12 (70.6)	12 (70.6)
Nha Trang				
Phuoc Tien (intervention)	14	11 (78.6)	7 (63.6)	10 (90.9)
Phuong Sai (delay)	21	11 (52.4)	7 (63.6)	6 (54.5)
Total n (%)	35	22 (62.9)	14 (63.6)	16 (72.7)
LONG AN				
Chau Thanh				
Long Tri (intervention)	4	4 (100.0)	4 (100.0)	4 (100.0)
An Luc Long (delay)	31	16 (51.6)	15 (93.8)	13 (81.3)
Total n (%)	35	20 (57.1)	19 (95.0)	17 (85.0)
Tan An				
Nhon Thanh Trung (Intervention)	6	6 (100.0)	5 (83.3)	5 (83.3)
Phuong 1 (delay)	6	5 (83.3)	2 (40.0)	2 (40.0)
Total n (%)	12	11 (91.7)	7 (63.6)	7 (63.6)
QUANG NAM				
Nui Thanh				
Tam Nghia (intervention)	19	15 (78.9)	13 (86.7)	10 (66.7)
Nui Thanh (delay)	19	11 (57.9)	8 (72.7)	7 (63.6)
Total n (%)	38	26 (68.4)	21 (80.8)	17 (65.4)
Tam Ky				
An My (intervention)	6	6 (100.0)	5 (83.3)	4 (66.7)
Tam Phu (delay)	24	12 (50.0)	9 (75.0)	7 (58.3)
Total n (%)	30	18 (60.0)	14 (77.8)	11 (61.1)
QUANG NINH				
Ha Long				
Hong Hai (intervention)	20	6 (30.0)	4 (66.7)	6 (100.0)
Ha Tu (delay)	14	10 (71.4)	5 (50.0)	9 (90.0)
Total n (%)	34	16 (47.1)	9 (56.3)	15 (93.8)
Van Don				
Đong Xa (intervention)	7	5 (71.4)	4 (80.0)	4 (80.0)
Ha Long (delay)	19	19 (100.0)	15 (78.9)	19 (100.0)
Total n (%)	26	24 (92.3)	19 (79.2)	23 (95.8)
THAI NGUYEN				
Phu Luong				
Yen Lac (intervention)	28	19 (67.9)	19 (100.0)	17 (89.5)
Đong Dat (delay)	21	14 (66.7)	13 (92.9)	12 (85.7)
Total n (%)	49	33 (67.3)	32 (97.0)	29 (87.9)
Song Cong				
Tan Quang (intervention)	16	15 (93.8)	12 (80.0)	12 (80.0)
Thang Loi (delay)	23	20 (87.0)	19 (95.0)	20 (100.0)
Total n (%)	39	35 (89.7)	31 (88.6)	32 (91.4)
THANH HOA				
Dong Son				
Đong Minh (intervention)	9	8 (88.9)	6 (75.0)	6 (75.0)
Đong Tien (delay)	10	10 (100.0)	7 (70.0)	6 (60.0)
Total n (%)	19	18 (94.7)	13 (72.2)	12 (66.7)
Quang Xuong				
Quang Ngoc (intervention)	22	21 (95.5)	17 (81.0)	17 (81.0)
Quang Phong (delay)	12	12 (100.0)	11 (91.7)	11 (91.7)
Total n (%)	34	33 (97.1)	28 (84.8)	28 (84.8)
Total				
Intervention	244	190 (77.9)	156 (82.1)	159 (83.7)
Delay	266	185 (69.5)	139 (75.1)	137 (74.1)
Total n(%)	510	375 (73.5)	295 (78.7)	296 (78.9)

The participants in communes randomized to “Immediate” received SSM in period 1 and those participants in communes randomized to “Delayed” received the enhanced treatment as usual in period 1

n_0_: total eligible participants after pre-screening

n_1_: total participants recruited in the trial and completed the baseline assessment

n_2_: total participants completed the second assessment

n_3_: total participants completed third assessment

**Table 2:** Study sample for Period 2

Province District Commune	Consent at baseline in Period 1. n0	Baseline n1 (n1/n0*100%)	Follow-up 1 (after 1 month) n2 (n2/n0*100%)	Follow-up 2 (after 2 months) n3 (n3/n0*100%)
BEN TRE				
Ben Tre				
Son Dong (intervention)	29	24 (82.8)	19 (65.5)	17 (58.6)
Phuong 6 (delay)	3	2 (66.7)	1 (33.3)	2 (66.7)
Total n (%)	32	26 (81.3)	20 (62.50)	19 (59.4)
Giong Trom				
Phuoc Long (intervention)	12	11 (91.7)	9 (75.0)	10 (83.3)
Long My (delay)	9	4 (44.4)	2 (22.2)	2 (22.2)
Total n (%)	21	15 (71.4)	11 (52.4)	12 (57.1)
DA NANG				
Hoa Vang				
Hoa Nhon (intervention)	8	4 (50.0)	2 (25.0)	4 (50.0)
Hoa Tien (delay)	17	11 (64.7)	9 (52.9)	9 (52.9)
Total n (%)	25	15 (60.0)	11 (44.0)	13 (52.0)
Thanh Khe				
Chinh Gian (intervention)	16	13 (81.3)	11 (68.8)	12 (75.0)
Thanh Khe Tay (delay)	8	2 (25.0)	2 (25.0)	1 (12.5)
Total n (%)	24	15 (62.5)	13 (54.2)	13 (54.2)
KHANH HOA				
Dien Khanh				
Dien An (intervention)	9	1 (11.1)	0 (0.0)	1 (11.1)
Dien Dien (delay)	8	6 (75.0)	4 (50.0)	6 (75.0)
Total n (%)	17	7 (41.2)	4 (23.5)	7 (41.2)
Nha Trang				
Phuoc Tien (intervention)	11	8 (72.7)	4 (36.4)	4 (36.4)
Phuong Sai (delay)	11	3 (27.3)	0 (0.0)	3 (27.3)
Total n (%)	22	11 (50.0)	4 (18.2)	7 (31.8)
LONG AN				
Chau Thanh				
Long Tri (intervention)	4	4 (100.0)	3 (75.0)	3 (75.0)
An Luc Long (delay)	16	8 (50.0)	7 (43.8)	7 (43.8)
Total n (%)	20	12 (60.0)	10 (50.0)	10 (50.0)
Tan An				
Nhon Thanh Trung (intervention)	6	3 (50.0)	1 (16.7)	1 (16.7)
Phuong 1 (delay)	5	3 (60.0)	2 (40.0)	2 (40.0)
Total n (%)	11	6 (54.5)	3 (27.3)	3 (27.3)
QUANG NAM				
Nui Thanh				
Tam Nghia (intervention)	15	10 (66.7)	8 (53.3)	7 (46.7)
Nui Thanh (delay)	11	7 (63.6)	7 (63.6)	6 (54.6)
Total n (%)	26	17 (65.4)	15 (57.7)	13 (50.0)
Tam Ky				
An My (intervention)	6	5 (83.3)	3 (50.0)	3 (50.0)
Tam Phu (delay)	12	6 (50.0)	4 (33.3)	4 (33.3)
Total n (%)	18	11 (61.1)	7 (38.9)	7 (38.9)
QUANG NINH				
Ha Long				
Hong Hai (intervention)	6	5 (83.3)	2 (33.3)	2 (33.3)
Ha Tu (delay)	10	7 (70.0)	6 (60.0)	6 (60.0)
Total n (%)	16	12 (75.0)	8 (50.0)	8 (50.0)
Van Don				
Đong Xa (intervention)	5	4 (80.0)	4 (80.0)	4 (80.0)
Ha Long (delay)	19	14 (73.7)	12 (63.2)	9 (47.4)
Total n (%)	24	18 (75.0)	16 (66.7)	13 (54.2)
THAI NGUYEN				
Phu Luong				
Yen Lac (intervention)	19	18 (94.7)	17 (89.5)	16 (84.2)
Đong Dat (delay)	14	10 (71.4)	9 (64.3)	8 (57.1)
Total n (%)	33	28 (84.8)	26 (78.8)	24 (72.7)
Song Cong				
Tan Quang (intervention)	15	11 (73.3)	11 (73.3)	11 (73.3)
Thang Loi (delay)	20	16 (80.0)	13 (65.0)	11 (55.0)
Total n (%)	35	27 (77.1)	24 (68.6)	22 (62.9)
THANH HOA				
Dong Son				
Đong Minh (intervention)	8	6 (75.0)	6 (75.0)	6 (75.0)
Đong Tien (delay)	10	5 (50.0)	5 (50.0)	5 (50.0)
Total n (%)	18	11 (61.1)	11 (61.1)	11 (61.1)
Quang Xuong				
Quang Ngoc (intervention)	21	18 (85.7)	17 (81.0)	17 (81.0)
Quang Phong (delay)	12	8 (66.7)	6 (50.0)	8 (66.7)
Total n (%)	33	26 (78.8)	23 (69.7)	25 (75.8)
Total				
Intervention	190	145 (76.3)	117 (61.6)	118 (62.1)
Delay	185	112 (60.5)	89 (48.1)	89 (48.1)
Total n(%)	375	257 (68.5)	206 (54.9)	207 (55.2)

The participants in communes randomized to “Immediate” discontinued SSM in period 2. These participants retained a copy of the workbook which they could use alone if desired, but received no formal coaching. Those participants in communes randomized to “Delayed” received SSM in period 2

n0: total participants recruited in the trial and completed the baseline assessment in period 1

n1: total participants completed the baseline assessment in period 2

n2: total participants completed the second assessment in period 2

n3: total participants completed third assessment in period 2

For ethical purposes, participants in the control (delayed intervention) group received the SSM intervention in Period 2 of the study, after approximately 4 months. Thus this study consists of a two-period modified stepped-wedge design, where it departs from a typical stepped-wedge design in that the immediate intervention group no longer received the intervention during Period 2 of the study (Fig. 1). The inclusion of Period 2 in the study is primarily for ethical reasons to ensure access by all study participants to evidence-based depression care in a context with minimal treatment availability with data collected for secondary analysis. Uncertainty regarding the flow of funds from government to the funding agency meant we were concerned with the feasibility of implementing Period 2 of the study. Therefore the study was powered to have the primary analysis to estimate intervention effects using Period 1 only [9], which is a simpler clustered randomized clinical trial (RCT) design. We conducted the secondary analysis that included the outcome data from Period 2 and additionally used the comparison between Period 1 and Period 2 for estimating SSM intervention effects. The higher amount of missing data in Period 2 also supports considering this supplemental analysis as a secondary analysis.

### Intervention and control

The treatment condition is SSM for depression, an intervention that is based on CBT principles and combines bibliotherapy, where a patient works through a structured work book, with coaching support by a non-specialist provider [7]. In Western contexts, SSM has been found to have a similar effect size to psychotherapy for depression interventions [10]. The SSM model used in this trial consists of providing the patient with the *Antidepressant Skills Workbook (ASW*) [11], which was developed by mental health specialists in Canada and was validated for cultural acceptability in Vietnam through a pilot study [8]. The ASW introduces patients to depression symptoms, describes options for treatment and includes guidance in three ‘antidepressant skills’ [11] (Table 3).

**Table 3:** Components of the An*tidepressant Skills Workbook* (Bilsker and Patterson, 2009)

Antidepressant skills	Activities
1. Reactivating your life	Identifying activities (e.g. self-care, social involvement), setting realistic goals, implementing and reviewing goals
2. Thinking realistically	Identifying depressive thoughts and their contribution to low mood, learning to challenge depressive thoughts and practicing realistic thinking
3. Solving problems effectively	Identifying problems and actions to solve them, develop and evaluate an action plan

In Vietnam, providers who have received no specialized training in mental health included primary care staff, social workers and social collaborators. Prior to study recruitment, providers were given enhanced training about depression to supplement the minimal training they routinely receive in mental health. Primary care staff were trained to administer the SRQ-20 and the World Health Organization’s Disability Assessment Scale 2.0 (WHODAS 2.0) and to refer patients scoring > 7 to social workers and social collaborators. Each commune has one designated qualified social worker, who has many responsibilities working on a broad portfolio for the Ministry of Labour, Invalids and Social Affairs (MOLISA). Because the social worker has limited time for service provision, social collaborators, who are lay social workers based in the community, support families and provide services. Qualified social workers have completed a four-year Bachelor of Social Work degree while the training and experience of social collaborators varies greatly. Social collaborators may be recruited to their role due to existing community involvement and leadership (e.g. as village care workers, Red Cross volunteers or Women’s Union staff). Social collaborators do not receive a monthly salary but may be provided a stipend for specific tasks, including screening for depression and delivery of SSM. A provincial-level social worker supervised social collaborators in the delivery of SSM. Social collaborators were selected to deliver the intervention in consultation with staff at MOLISA, who, at the time of the study, prioritized the use of social collaborators to deliver community-based mental health services.

Social collaborators received 3 days of training by the study team, a psychiatrist from the provincial psychiatric hospital and district-level representatives of both the health and social services sectors. Training components included screening for depression using the SRQ-20 and in the delivery of the coaching intervention. They participated in 3 days of training, the first of which was a classroom session to introduce them to depression symptoms, etiology, screening and the principles and practice of the ASW. The next 2 days of coaching were spent in the field practicing learned skills with the supervision of the trainers. During the two-month course of the intervention, each social collaborator received two visits from a provincial-level social worker during their coaching session with patients to provide supervision and support, and to assess fidelity.

As part of the SSM intervention social collaborators provided one-on-one coaching on the use of the ASW at the home of participants over the course of two-months. Six to ten social collaborators per commune delivered the intervention, with numbers varying by commune size. Coaching sessions took place every 2 weeks, during which the social collaborator consulted with the patient on progress, reviewed the concepts in the ASW, and helped to create a plan for the subsequent 2 week period.

The control condition was enhanced treatment as usual, which consisted of treatment as usual plus provision of an adapted leaflet based on the “Understanding Depression” pamphlet by Beyond Blue (www.beyondblue.org), providing participants with information about depression, its symptoms, risk factors and approaches to care. Because of the low recognition of depression in primary care in Vietnam and limited resources, treatment as usual was likely to mean minimal or no treatment following screening. Participants in the control group, with the exception of those with severe depression or suicidal ideation, were not referred to secondary care at this stage but were free to access additional services on their own accord.

### Participants

**Fig. 2:**
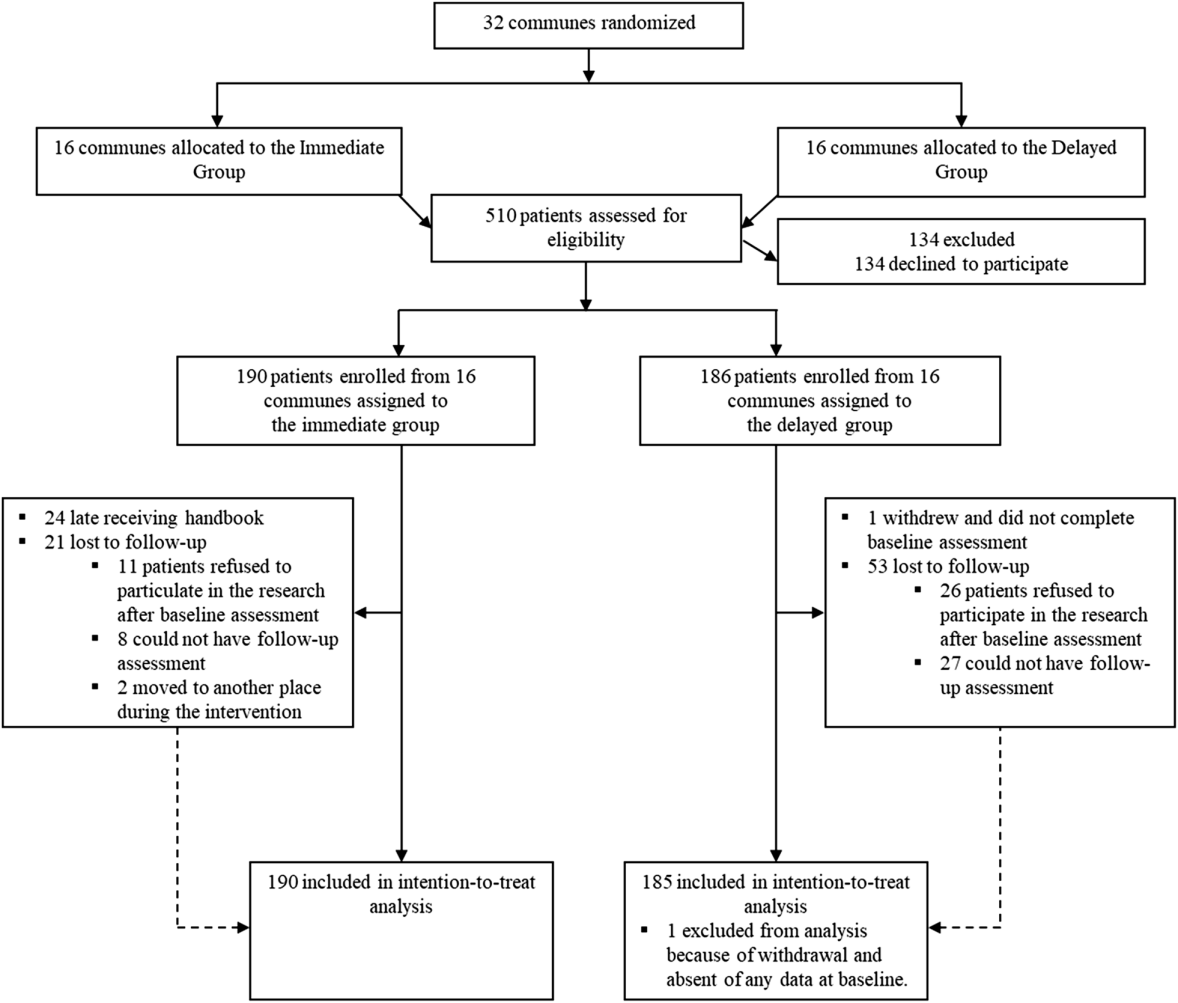
CONSORT flow diagram

Figure 2 displays the CONSORT flow diagram of the trial. Recruitment procedures have been described in more detail elsewhere [9]. Adults 18 years and older were recruited in primary care and community-based settings in the study communes. Primary care providers at Commune Health Stations (CHSs) screened patients attending non-emergency consultations, while community-based social collaborators screened community members thought to be at risk of depression, including those who were recently bereaved or had seriously ill family members, had experienced marital breakdown, had experienced financial loss or bankruptcy, or were reluctant to leave their home or to engage in normal social or work activity. Social collaborators are embedded in communities and are familiar with community members, meaning that they are often aware when people experience potential risk-factors for depression.

Inclusion criteria for the study were: (1) a score of > 7 on the SRQ-20, indicating probable depression caseness [12]; (2) completion of written informed consent and agreement to complete study outcome measures. Exclusion criteria were: (1) cognitive impairment based on patient history; (2) symptoms of psychosis, severe depression or suicidal ideation; (3) impaired vision or hearing; (4) illiteracy.

In cases of severe mental illness, including presence of suicidal ideation, patients were referred to tertiary psychiatric facilities. In total 13 patients with severe depression were referred to hospitals.

As randomization occurred at the commune level, participants in the immediate intervention and control (delayed intervention) groups accessed services in different locations, minimizing the risk of contamination.

Ethical approval was obtained from the Research Ethics Board of Simon Fraser University in Vancouver, Canada [#2016s0604] and from the Institutional Review Board of the Institute of Population, Health and Development (PHAD) in Hanoi, Vietnam [2016/PHAD/MAC-FI-AD-01-01].

### Instruments

The SRQ-20, a 20-item scale designed by the WHO to screen for psychological disturbance, including depression [13], was used to screen participants and to assess change in depression symptoms. Each item of the SRQ-20 can be scored at 0 or 1, with 1 indicating that the symptom was present during the past month. The SRQ-20 was selected based on a review of depression measures that have been previously used and validated in Vietnamese populations, where it was found to be the most appropriate measure [12]. It has previously been found to be valid and appropriate for use by lay health workers in LMICs [14]. The appropriateness of the SRQ-20 was further assessed during the feasibility study. There is no universal cut-off for the SRQ-20, but previous studies validating and using the SRQ-20 in Vietnam identified a score of > 7 as the appropriate cut-off to indicate probable depression caseness among Vietnamese adults [13].

The World Health Organization Disability Assessment Scale version 2.0 (WHODAS 2.0) is a generic instrument that is used to assess disability across six domains: cognition, mobility, self-care, getting along, life activities, and participation. Each item has five potential scores—“none” [1], “mild” [2] “moderate” [3], “severe” [4] and “extreme” [5]—which are summed to produce a score for each domain. The WHODAS 2.0 assesses changes in disability and was also used in the feasibility study. It has been identified as an appropriate measure of disability for use in community-based populations [15] and has been administered by lay interviewers in LMIC contexts [16].

### Randomization and masking

Randomization was conducted using permuted blocks to conceal allocation and was stratified according to district. The randomization sequence was developed and controlled by one individual (CHG at Simon Fraser University) not involved in data collection for the study to ensure fidelity.

The nature of the SSM intervention did not allow for full blinding. Outcome assessors, who were research staff in Hanoi not otherwise involved in the study, were blinded and conducted one- and two-month assessment interviews in Period 1 with participants by telephone. The assessors were provided with telephone numbers for participants and had no knowledge of patient location or whether they were allocated to the immediate or delayed intervention group. Participants were told not to disclose to assessors whether they were part of the immediate or delayed intervention group.

### Outcomes

The primary outcome for this trial is the effect of SSM on change in depression scores, based on the proportion of participants in the intervention group with SRQ-20 scores > 7 as compared to the control group at two-months.

As secondary outcomes, the absolute change in SRQ-20 and WHODAS 2.0 were examined.

The study included six outcome assessment points. In Period 1, outcome measures were collected at baseline, at 1 month and at 2 months for both intervention and control groups. In Period 2, outcome measures were collected from the delayed intervention group (Period 1 control group) and the post-intervention group (Period 1 intervention group) at baseline in Period 2, at 1 month and at 2 months after baseline in Period 2.

The trial’s Data Monitoring Committee (DMC), composed of representatives from Simon Fraser University (CHG as Chair), PHAD (NKC), and the Hanoi University of Public Health (HUPH), provided oversight on trial safety. The DMC’s mandate was consistent with SPIRIT guidelines [17] and was independent of the study funder and had no competing interests. The committee met three times during the trial and identified no concerns regarding safety or adverse events.

### Statistical analysis

The sample size required for this trial was calculated [9]. In summary, it was assumed that each commune would recruit an average of eight participants with a follow-up period of 2 months after baseline and an effect size of 0.4 for the SRQ-20, where the effect size was estimated as the ratio of the minimum clinically important difference divided by the standard deviation at baseline across all study participants. The calculation shows that for a type I error rate of 0.05, a power of at least 80%, and an intracluster correlation coefficient (ICC) of 0.05, a total of 268 subjects were needed. Our intention-to-treat (ITT) sample of 375 subjects in total from 32 clusters exceeded this calculated sample size to account for attrition and missed visits.

The primary analysis was ITT using outcome data measured at months one and two in Period 1 for all participants in randomly allocated communes from a clustered RCT. Our study was powered for this primary analysis. This primary analysis uses Period 1 data only and has the advantage of being unaffected by the higher amounts of missing data in Period 2. The analysis was based on the individual patient-level data, rather than on the commune-level summarized data as tentatively suggested in Murphy et al. 2017 [9]. The individual-level analysis had the advantages of exploiting the full richness of the individual-level data and utilizing the individual-level covariates and observed outcome values to account for missing outcome values. The individual-level analysis is also natural to account for missing data issues in our study because no communes were lost to follow-up in this study and all missing data occurred at the individual patient level. The individual participants’ binary depression outcomes (SRQ-20 > 7) were analyzed using logistic mixed effect regression models and their continuous outcomes (SRQ-20 and WHODAS 2.0 scores) using linear mixed effect regression models. These models belong to the Generalized Linear Mixed-effects models (GLMMs) that are the most efficient and recommended statistical methods for analyzing clustered and longitudinal clinical trial data [18]. GLMMs have been widely used for conducting ITT analysis in such trials with missing data [19]. The methods can account for data missing at random without the need to model why data are missing or perform explicit imputations of the missing values [20]. These models included the outcome at months 1 and 2 in Period 1 as the response variable, indicator variables for visits (for secular trend), indicators for SSM intervention effects at months 1 and 2, and baseline score as fixed effects. Additionally, these models included random effects for communes (the clusters) and for participants nested within communes to account for random variation between communes and between participants within the same commune. The sandwich estimators for GLMMs [20] were used to compute empirical standard errors that are robust to model specifications. An unstructured variance–covariance matrix was used to model the within-subject error variance–covariance structure for continuous outcomes. Results are reported in Table 3 as adjusted OR (odds ratio for having SRQ-20 > 7 when a subject receives SSM relative to when the same subject receives the treatment as usual) for binary outcome (SRQ-20 > 7) and as Δ (the adjusted difference in the mean value of SRQ-20 or WHODAS2.0 when a subject receives SSM relative to when the same subject receives the enhanced treatment as usual). These intervention effect estimates, 95% CIs and p-values are obtained from the above logistic mixed effect models for SRQ-20 > 7 and the linear mixed effects models for SRQ-20 and WHODAS2.0 at month 1 and month 2 in Period 1, adjusting for random effects, the baseline outcome values at Period 1 and dummy variables for secular trends at follow-up visits. Standardized effect sizes were calculated for continuous scores, dividing the adjusted mean differences by the SDs across all participants at baseline.

To evaluate the robustness of the results to alternative assumptions regarding missing data, sensitivity analyses were conducted via (1) using selection models [21, 22] and (2) adjusting analysis for baseline covariates potentially predictive of missing data for the primary and secondary outcomes. We also conducted a secondary GLMM analysis using data from both Period 1 and Period 2. The GLMMs described above for modelling Period 1 data only were expanded to include additional indicator variables for visits in Period 2 to account for secular trends and for estimating treatment carryover effects [23]. All the analysis was conducted in SAS version 9.4 except that the sensitivity analysis to selection models was conducted in the package isni in R 3.4 [21].

## Results

Between July 2016 and November 2017 a total of 32 communes from 16 districts across eight provinces in Vietnam were enrolled in the study. Two communes in each district were randomly selected from a list of communes with homogenous population characteristics. Thirty-two communes with a total of 376 participants were randomly assigned to receive the SSM intervention in Period 1 (16 communes with a total of 190 participants in the immediate intervention group) or enhanced treatment as usual in Period 1 (16 communes with a total of 186 in the delayed intervention group, Fig. 1, Tables 1 and 2). The baseline characteristics were well balanced in the two randomized groups (Table 4). Chi square tests for categorical variables and two-sample t-tests were used to compare the distributions of baseline variables between two randomization groups; no group difference is found to be statistically significant at the 0.05 level for any baseline variables listed in Table 4.

**Table 4:** Baseline Characteristics in Period 1

Categorical variables	Description	Immediate (n = 190)	Delayed (n = 186)	Total (n = 376)
		n (%)	n (%)	n (%)
Living situation	Independent in the community	187 (98.4)	184 (98.9)	371 (98.7)
	Assisted in the community	1 (0.5)	0 (0.0)	1 (0.3)
	Hospitalized	0 (0.0)	1 (0.5)	1 (0.3)
	Missing	2 (1.1)	1 (0.5)	3 (0.8)
Sex	Male	32 (16.8)	26 (14.0)	58 (15.4)
	Female	158 (83.2)	159 (85.5)	317 (84.3)
	Missing	0 (0.0)	1 (0.5)	1 (0.3)
Marital status	Never married	8 (4.2)	13 (7.0)	21 (5.6)
	Current married	149 (78.4)	129 (69.4)	278 (73.9)
	Separated	2 (1.1)	11 (5.9)	13 (3.5)
	Divorced	9 (4.7)	5 (2.7)	14 (3.7)
	Widowed	20 (10.5)	25 (13.4)	45 (12.0)
	Cohabiting	2 (1.1)	1 (0.5)	3 (0.8)
	Missing	0 (0.0)	2 (1.1)	2 (0.5)
Working status	Self employed	53 (27.9)	61 (32.8)	114 (30.3)
	Farmer	71 (37.4)	70 (37.6)	141 (37.5)
	Housewife	27 (14.2)	28 (15.1)	55 (14.6)
	Retired	4 (2.1)	6 (3.2)	10 (2.7)
	Unemployed (health reasons)	10 (5.3)	4 (2.2)	14 (3.7)
	Unemployed (other reasons)	3 (1.6)	2 (1.1)	5 (1.3)
	Other jobs	1 (0.5)	1 (0.5)	2 (0.5)
	Paid work	21 (11.1)	13 (7.0)	34 (9.0)
	Missing	0 (0.0)	1 (0.5)	1 (0.3)

Continuous variables		Immediate (n = 190)	Delayed (n = 186)	Total
SRQ20	N (Nmiss)	190 (0)	185 (1)	375 (1)
	Mean (SD)	10.97 (2.46)	10.68 (2.22)	10.82 (2.34)
	Min, max	8, 18	8, 17	8, 18
	Q1, Median, Q3	9, 10, 12.75	9, 10, 12	9, 10, 12
WHODAS2.0	N (Nmiss)	190 (0)	185 (1)	375 (1)
	Mean (SD)	26.92 (7.62)	26.70 (8.90)	26.81 (8.26)
	Min, Max	14, 57	12, 51	12, 57
	Q1, Median, Q3	21, 26, 31	21, 25, 32	21, 26, 31

Totals may not equal 100 due to rounding

The assessment of the intervention effects on the outcome variables used the intention-to-treat (ITT) analysis. All randomly assigned 32 communes were followed up to the end of the trial (i.e., no loss of follow-up for communes) and were included in the analysis. The analysis excludes one participant who was randomized to the delayed intervention group, withdrew and did not provide any data at baseline. The ITT analysis is not affected by including or excluding this patient, who provided no data. Thus ITT analysis was conducted on the remaining 375 participants (190 in the immediate intervention group and 185 in the delayed intervention group). The percentage of participants completing the outcome assessment in the delayed intervention group receiving the enhanced treatment as usual with depression (SRQ20 > 7) is shown in Table 5. Mixed-model analysis shows that the adjusted odds ratio of having depression between receiving the SSM intervention and receiving the control was 0.47 (p = 0.0038), meaning that receiving the intervention reduces the odds of having depression by 53%, as compared with receiving the control after 1 month of treatment.

**Table 5:** Primary analysis of depression and disability outcomes in the intervention and control groups at 1 month and 2 months (Period 1)

	Period 1	Unadjusted mean		Adjusted difference (primary analysis)	*p* value	Adjusted difference (robustness checking)	p-value
		Immediate intervention (n0 = 190)	Delayed intervention (n0 = 185)				
Primary outcome							
SRQ-20 > 7 (%)	Baseline	100% (190/190)	100% (185/185)	–	–	–	–
	1 month	57.1% (89/156)	71.2% (99/139)	OR = 0.47 95% CI (0.28, 0.78)	0.0038	OR = 0.39 95% CI (0.23, 0.64)	0.0002
	2 month	26.4% (42/159)	42.3% (58/137)	OR = 0.42 95% CI (0.28, 0.63)	< 0.0001	OR = 0.33 95% CI (0.21, 0.52)	< 0.0001
Secondary outcomes							
SRQ-20	Baseline	11.0 (2.5)	10.7 (2.2)	–	–	–	–
	1 month	8.4 (4.7)	9.9 (4.3)	Δ = − 1.76 95% CI (− 2.72, − 0.79)	0.0004	Δ = − 1.84 95% CI (− 2.81, − 0.86)	0.0002
	2 month	5.2 (4.6)	7.5 (4.5)	Δ− 2.42 95% CI (− 3.38, − 1.40)	< 0.0001	Δ = − 2.50 95% CI (− 3.47, − 1.51)	< 0.0001
WHODAS2.0	Baseline	26.9 (7.6)	26.7 (8.9)	–	–	–	–
	1 month	22.9 (9.6)	24.5 (9.3)	Δ = − 1.86 95% CI (− 3.82, 0.06)	0.059	Δ = − 1.93 95% CI = (− 3.85, -0.02)	0.047
	2 month	18.3 (7.4)	21.0 (8.3)	Δ = − 2.63 95% CI (− 4.32, − 0.99)	0.002	Δ = − 2.68 95% CI = (− 4.34, − 1.02)	0.0016

Data are mean (n/N) for the binary primary outcome and mean (SD) for the continuous secondary outcomes, where n is the number of participants with SRQ-20 > 7 and N is the number of participants completing the outcome assessment and N is the same for all three outcomes in the study. The last two columns present results from robustness checking that additionally adjusted for the baseline covariates (gender, working status, living status and marital status)

*OR* odds ratio, Δ mean difference

Table 5 also shows the percentage of participants in the control and delayed groups completing the outcome assessment with depression (SRQ20 > 7) at 2 months after baseline in Period 1Mixed model analysis shows that the adjusted odds ratio of having depression between receiving the SSM intervention and receiving the control was 0.42 (p < 0.0001, Table 5), meaning that receiving the intervention reduces the odds of having depression by 58%, as compared with receiving the control after 2 months of treatment.

**Fig. 3:**
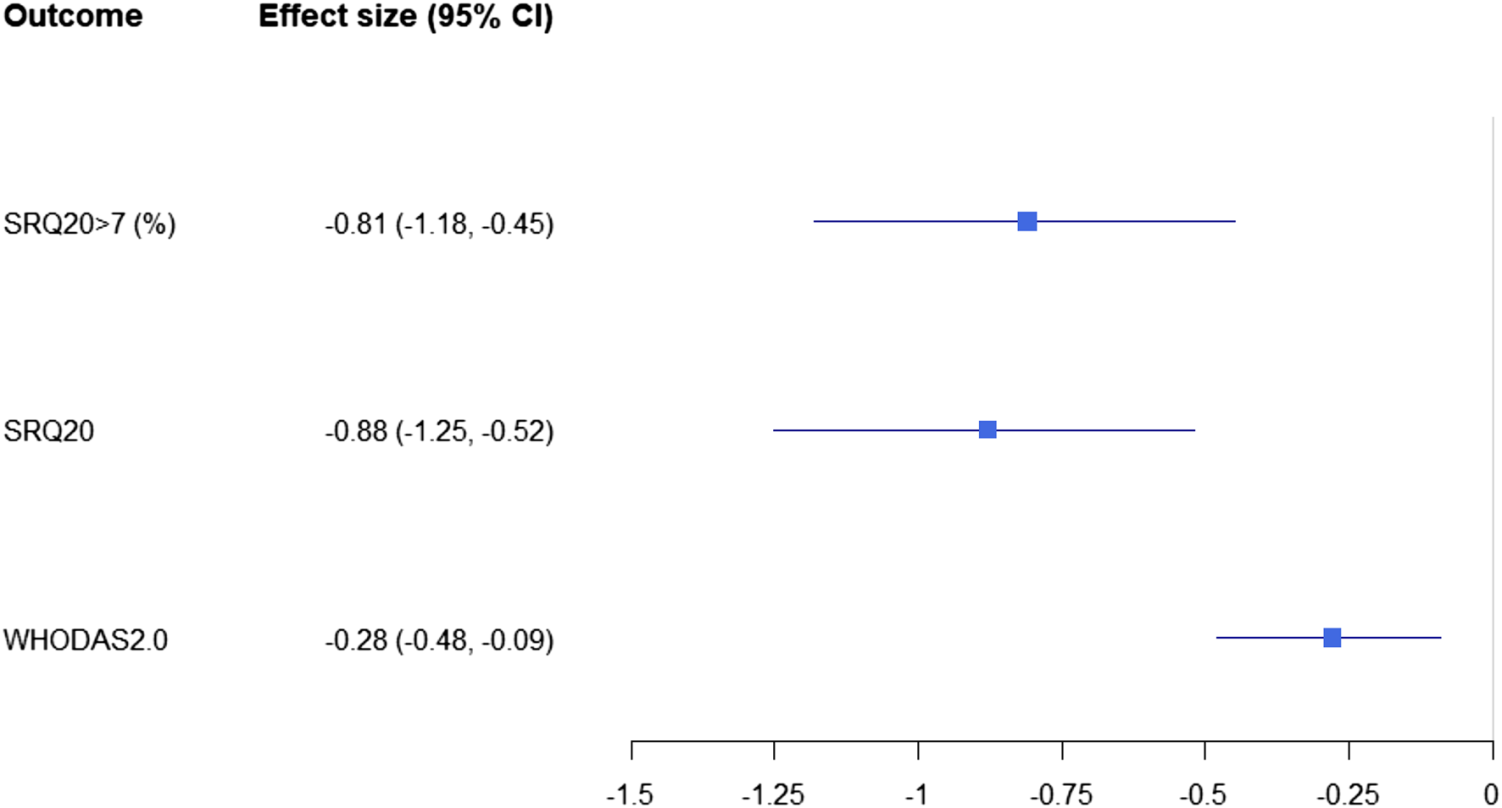
The overall effect sizes and 95% cis in Period 1

Note: The forest plot displays the overall effect sizes pooled at 1 month and 2 months after intervention initiation in period 1, and 95% CIs for these overall effect sizes.

Regarding the secondary outcomes, the results of the ITT analysis are shown in Table 5. The intraclass correlation coefficient (ICC) for the outcome SRQ-20 is estimated as 0.04. The estimated effect size and the ICC value for SRQ-20 show that our study is adequately powered (i.e., > 80% power at a type I error rate of 0.05). Table 5 also shows the reduction of WHODAS scores at 1 month and 2 months after treatment in Period 1. The ICC is estimated as 0.07 for the WHODAS 2.0. Finally, none of the primary and secondary outcomes show evidence for differential intervention effects at 1 month and 2 months after intervention initiation in period 1, with p-values for testing homogenous intervention effects at these two time points being 0.72, 0.20, 0.34 for SRQ-20 > 7, SRQ-20 and WHODAS 2.0, respectively. Thus we fit simpler models assuming homogeneous intervention effect at the two time points to produce overall effect estimates that are more precise. Figure 3 plots the overall intervention effects pooled at these two time points for the primary and secondary outcomes. Effect size in Fig. 3 for the binary outcome SRQ > 7 is calculated as the adjusted between-group differences in the logarithm of odds of having depression. Effect sizes for the two continuous outcomes (SQR20 and WHODAS2.0) are calculated as the adjusted between-group differences in the means of each outcome, divided by the SD of the outcome at baseline across all participants. Negative values of effect sizes mean beneficial SSM treatment effect for all three outcomes. A small effect size is [− 0.5, − 0.2]; medium is [− 0.8, − 0.5]; large is < − 0.8^8^.

There was a comparable and moderate amount of missing data in both groups in Period 1 (Table 6). Thus there are reasons to believe that the analysis conducted above is likely insensitive to the assumption of data missing at random (MAR). The following analyses were conducted to formally quantify the robustness of our primary findings to alternative missing data assumptions. First selection models were used that permit the missingness probability to depend on the unobserved outcome values after conditioning on the observed data and then we computed an Index of local Sensitivity to Non ignorable Missingness (ISNI) [21, 22]. The ISNI analysis results are reported in Table 6. For binary outcome SRQ-20 > 7, ISNI estimates the change in intervention effect estimates listed in the column “Adjusted Differences (Primary Analysis)” in Table 5 for a moderate size of nonrandom missingness where a patient with SRQ-20 > 7 has an increase of e^1^ = 2.7-fold in the odds of being observed relative to a patient with SRQ-20 ≤ 7, given that both participants have the same values of observed predictors for missingness (baseline outcome and covariate values, and most recently observed prior outcome value, visit dummy variables, randomization groups, communes, missingness status in prior visits). We then compute the tipping point (TP), which approximates the threshold size of nonrandom missingness required to change statistical significance results to nonsignificant (i.e. confidence interval cover the value of 1 for odds ratio), where the size of nonrandom missingness is described by the log odds ratio of being observed for a patient with SRQ-20 > 7 relative to a patient with SRQ-20 ≤ 7 and the same values on the aforementioned predictors for missingness. For continuous outcome SRQ-20 and WHODAS2.0, ISNI/SD in Table 6 estimates the change in intervention effect estimates for a moderate size of nonrandom missingness where a one-SD (standard deviation of the outcome) increase in the outcome is associated with an increase of e^1^ = 2.7-fold in the odds of being observed, conditioning on the same values of the aforementioned observed predictors for missingness. We also computed the TP, which approximates the threshold size of nonrandom missingness required to change statistical significance results, where the size of nonrandom missingness is described by the log odds ratio of being observed associated with one-SD increase in the outcome, conditioning on the same values on the aforementioned observed predictors for missingness.

**Table 6:** Sensitivity Analysis of primary results of group comparisons in period 1 to the assumption of data missing at random (MAR)

Month	Missing data percentage in Period 1		SRQ-20 > 7		SRQ-20		WHODAS2.0	
	Immediate	Delayed	ISNI	TP	ISNI/SD	TP	ISNI/SD	TP
1	17.8% (34/190)	24.8% (46/185)	0.03	4.4	0.28	2.8	0.52	0.2
2	16.3% (31/190)	25.9% (48/185)	0.01	23.4	0.21	6.8	0.44	2.3

Missing data percentage is Mean (n/N) where n is the number of participants completing the outcome assessment and N is the number of participants in the ITT analysis sample

TP (tipping point) = (1-effect estimate− 1.96*standard error)/ISNI [19, 20] for binary outcome SRQ-20 > 7 and TP (Tipping Point) = (Effect estimate − 1.96*standard error)*SD/ISNI [22, 24] for continuous outcome SRQ-20 and WHODAS 2.0

*ISN* index of sensitivity to non ignorability, *SD* standard deviation

The ISNI results in Table 6 show that the intervention effect for the primary outcome (SRQ-20 > 7) at month 2 remains statistically significant so long as the degree of nonrandom missingness is no larger than the tipping point (TP = 23.4, Table 6). A TP value of 23.4 means an extreme and unlikely scenario such that a patient with SRQ-20 > 7 has an increase of e^23.4^-fold (≈ 1.5 × 10^10^-fold) in the odds of being observed relative to a patient with SRQ-20 ≤ 7 and the same values on observed predictors for missingness. A TP value of this large size is not meant to capture the exact tipping point precisely, but merely means that one has to consider extreme cases of non-random missingness to find sensitivity. In fact, even when replacing all missing values for SRQ-20 > 7 with “No”, the beneficial intervention effect estimate remains statistically significant (p = 0.0063). The beneficial intervention effect also remains significant when replacing all missing values for SRQ-20 > 7 with “Yes” (p < 0.001). Thus we conclude the statistical significance of the intervention effect for the primary outcome SRQ-20 > 7 at Month 2 is robust to the violation of the MAR assumption. The magnitudes of nonrandom missingness at the tipping point for SRQ20 > 7 at month 1 and for the secondary outcomes are all relatively strong at the tipping point (Table 6), suggesting the robustness to moderate violations of the MAR assumption, except for the WHODAS 2.0 at month 1 for which a slight degree of nonrandom missingness (TP = 0.2, Table 6) could turn its statistical insignificance to become significant. Secondly baseline covariates were included in the GLMM outcome models and the intervention effects for the primary and secondary outcomes remain statistically significant with intervention effect estimates pointing to stronger beneficial treatment effects (Table 5 the last two columns).

The ITT analysis of outcome data from both Period 1 and Period 2 was also conducted. Table 7 presents the summary of the outcome data in Period 2 with mixed effects model estimation results presented in Table 8. The intervention effect in Table 8 refers to the pooled effects of SSM at 1 and 2 month after treatment initiation since there was no statistically significant difference in the intervention effects at these two time points. The carry-over effect refers to the lasting effect of the full B + S SSM treatment received in Period 1 on the outcome variables at Period 2 for participants in the immediate group who discontinued the full B + S SSM treatment in period 2. These effect estimates, 95% CIs, p-values are obtained from logistic mixed effect models for SRQ-20 > 7 and linear mixed effects models for SRQ-20 and WHODAS2.0 at month 1 and month 2 in Period 1 and baseline, 1 month and 2 Months in Period 2, with random effects for communes and for subjects nested within communes, adjusting for the baseline outcome values at Period 1 and dummy variables for secular trend in all follow-up visits. The complementary analysis shows similar beneficial intervention effect estimates as in primary analysis described above. This complementary analysis additionally estimates the effects of intervention effect carried over from Period 1 to Period 2 in the immediate intervention group. Specifically the carry-over effect refers to the lasting impact of the full SSM intervention (book and support—B + S) treatment received in Period 1 on the outcome values at Period 2 after discontinuing the full B + S treatment in the immediate intervention group. The analysis shows that the improvement in the participants’ condition in the immediate intervention group indeed persisted in Period 2 even if the full B + S treatment was stopped (Table 8). The carry-over effects are estimated to be 0.23 (p = 0.0035) for adjusted odds ratio of having depression (SRQ-20 > 7), − 3.0 (p = 0.0003) and − 4.4 (p = 0.0069) for adjusted mean differences in SRQ-20 and WHODAS2.0, respectively.

**Table 7:** Depression and disability outcomes in the intervention and control groups at 1 month and 2 months in Period 2

Period 2	Immediate intervention (N0 = 190)			Delayed intervention (N0 = 185)		
	Baseline (N = 145)	1 month (N = 117)	2 months (N = 118)	Baseline (N = 112)	1 month (N = 89)	2 months (N = 89)
Primary outcome						
SRQ-20 > 7 (%)	35.9% (52/145)	27.4% (32/117)	18.6% (22/118)	54.5% (61/112)	39.3% (35/89)	19.1% (17/89)
Secondary outcomes						
SRQ-20	6.1 (4.8)	5.4 (4.8)	3.7 (4.5)	8.6 (5.5)	6.4 (5.4)	4.1 (4.7)
WHODAS2.0	18.9 (7.9)	19.4 (9.8)	19.6 (9.1)	21.8 (9.5)	22.5 (10.2)	21.0 (10.0)

Data are mean (n/N) for the binary primary outcome and mean (SD) for the continuous secondary outcomes, where n is the number of participants with SRQ-20 > 7 and N is the number of participants completing the outcome assessment and N is the same for all three outcomes in the study

**Table 8:** Secondary analysis of study outcomes using data from both Period 1 and Period 2

	Intervention effect		Carryover effect	
	Adjusted difference (95% CI)	p-value	Adjusted difference (95% CI)	p-value
Primary outcome				
SRQ-20 > 7	OR = 0.41 95% CI (0.26, 0.62)	< 0.0001	OR = 0.23 95% CI (0.08, 0.61)	0.0035
Secondary outcome				
SRQ-20	Δ = − 2.11 95% CI (− 2.93, − 1.28)	0.0001	Δ = − 2.98 95% CI (− 4.60, − 1.36)	0.0003
WHODAS2.0	Δ = −2.32 95% CI (− 3.79, − 0.85)	0.002	Δ = −4.37 95% CI (−7.53, −1.21)	0.0069

*OR* odds ratio, Δ mean difference

## Discussion

The main results of this study demonstrate the effectiveness of the SSM intervention for reducing the symptoms of depression among adults with mild to moderate depression and the percentage of participants scoring > 7 on the SRQ-20 in community-based settings in Vietnam. The secondary outcomes suggest that SSM may reduce disability. The effectiveness of the SSM intervention, delivered in community-based settings by minimally trained lay social workers, employing principles of “task-sharing”, has important implications for improving availability of and access to depression care in the Vietnamese context. This study also contributes to the broader global mental health evidence base, as it demonstrates that community-based task-sharing interventions for depression may be effective and viable across low-resource contexts.

The methodological design employed in this study may also be of interest in other LMIC contexts. Given the minimal availability of depression care in the Vietnamese context, ensuring that the control group had access to the intervention following Phase 1 data collection was ethically necessary. This approach may be considered for ethical purposes in similar settings where mental health care is limited.

### Clinical significance and importance

In addition to the statistically significant reduction of symptoms of depression experienced by study participants who received SSM, the clinical significance of SSM should be considered. Clinical significance has been a subject of debate and several approaches to its measurement and interpretation have been used [24]. Response, remission, recovery, and functional impairment, among other factors, have been considered as measures of clinical significance for depression [24]. In this study, participants experienced a reduction in depression symptoms based on the SRQ-20. The carry-over effect noted in Period 2, when participants in the Phase 1 intervention group no longer received the active intervention, suggests that a significant proportion of the immediate intervention group may have met the criteria for remission as defined by the Macarthur Foundation Task Force [25]. Clinical significance of SRQ-20 change scores is not well-established, and evidence regarding interpretation of clinical significance using the SRQ-20 is extremely limited. This study was powered for medium clinical significance with effect size at 0.5, with > 0.8 considered a large effect size [9]. The analysis shows an effect size of − 1.03. The results for the primary outcome also show that the odds of having SRQ > 7 is reduced by 58% for a participant receiving SSM compared to receiving the control. While the results related to clinical significance are promising, additional research on the clinical significance of SRQ-20 scores in the Vietnamese context would further clarify these results. Disability, as measured by the WHODAS 2.0 in this study, is considered an appropriate secondary indicator of clinical significance [24, 25]. The results of the secondary analysis in this study show a small effect size of approximately 0.3.

The current study examined the effectiveness of SSM in reducing depressive symptoms and disability in participants experiencing mild to moderate depression. Though some patients with severe depression receive treatment in tertiary care settings in Vietnam, a substantial gap in availability of care for severe depression remains. Psychological treatment offered in combination with pharmacotherapy is recommended in clinical depression guidelines regardless of severity [26]. Offered in combination with antidepressant medications, SSM may also constitute effective and appropriate care for patients with severe depression in Vietnam. Delivery of combined treatment, however, will require continued strengthening of the mental health system, including improving the availability of antidepressant medications and practitioner clinical skills in community-based settings.

### Policy, implementation and scale-up implications

A core characteristic of the study intervention is its delivery by non-specialist providers via task-sharing. The delivery of care using task-sharing methods in the context of limited human and financial resources has been implemented in other areas such as HIV [27] and non-communicable diseases [28]. Task-sharing for mental health services is identified by the 2018 *Lancet Commission on Global Mental Health and Sustainable Development* as a key innovation to be scaled-up in LMICs [3]. The evidence base for the effectiveness of task-sharing in mental health is growing. A 2017 review of randomized trials in LMICs showed that lay health workers helped reduce the burden of common mental disorders including depression using a variety of psychological techniques, including psycho education and goal setting [5]. Randomized trials in India [29], Brazil [30], and Zimbabwe [31] have suggested that interventions delivered by lay health workers for common mental disorders are effective. This study contributes to the accumulating evidence on the effectiveness of task-sharing interventions for depression in an LMIC setting, suggesting that both primary care providers and social collaborators can offer an effective psychosocial intervention for depression. This model can help to fill a critical gap in care for mild to moderate depression in Vietnam.

The task-sharing model used in this study involved both the health and social services sectors at the community level (village care workers, village Red Cross workers and village women’s union staff, in this context called ‘social collaborators’). Due to low help-seeking and awareness about depression among patients in Vietnam, this model may help to improve public knowledge and reach. The involvement of both the health and social services sectors in the intervention reported here contributes to more coordinated rather than siloed approaches to mental health care provision, though inter-ministerial collaboration remains a challenge in Vietnam.

Evidence suggests that task-sharing models can be cost-effective for health systems [32, 33]. This study did not include a cost-effectiveness analysis, and further exploration of the cost-effectiveness of SSM in the Vietnamese context will be undertaken as part of a follow-up study.

For reasons noted in the section of “Study Design”, the primary analysis of this clinical trial, as reported in Table 5, uses only data in period 1, which forms a simpler and standard clustered randomized clinical trial (RCT) design. The secular trend of the outcomes for the delayed group in period 1, shown in Table 5, captures the natural course of depression, including possible remission and recovery in the absence of intervention, and demonstrates the value of conducting a randomized clinical trial to disentangle the natural secular trend from true intervention effect. In the secondary analysis that uses both periods of this two-period modified stepped-wedge design, more advanced statistical modeling developed for this type of data is employed to distentagle the natural secular trend from intervention effect [21].

## Conclusions

The government of Vietnam has prioritized the enhancement of community-based care for depression. MOLISA has been an actively involved partner in the work reported here, contributing matched funding for the current study and engaging in ongoing communication with the study team. The engagement of MOLISA through this study is vital for the potential scale-up of the SSM model in Vietnam. The results of this study have the potential to directly contribute to evidence-informed policy making for mental health in Vietnam. A follow-up study, funded by the Canadian Institutes of Health Research (CIHR), will enable us to study, in real time, factors influencing the implementation of a national scale-up of SSM within the dynamic policy context of Vietnam.

## Data Availability

Study datasets are currently being stored in Simon Fraser University’s RADAR research data repository. Access to the data can be requested from the study authors.

## Abbreviations

ASW: *Antidepressant skills workbook*
B + S: Book and support
CBT: Cognitive behavioural therapy
CIHR: Canadian Institutes of Health Research
DMC: Data Monitoring Committee
GLMM: Generalized linear mixed-effects model
HUPH: Hanoi University of Public Health
ICC: Intraclass Correlation Coefficient
ISNI: Nonignorable missingness
ITT: Intention-to-treat
LMICs: Low and middle-income countries
MAR: Missing at random
MOLISA: Ministry of Labour, Invalids and Social Affairs
PHAD: Institute of Population, Health and Development
RCT: Randomized control trial
SRQ-20: Self-reporting questionnaire 20-item
SSM: Supported self-management
TP: Tipping point
WHODAS 2.0: World Health Organization’s Disability Assessment Scale 2.0
